# Supplementary material for: APOB CRISPR-Cas9 Engineering in Hypobetalipoproteinemia: A Promising Tool for Functional Studies of Novel Variants
Source: Int J Mol Sci. 2022 Apr 13;23(8):4281. doi: 10.3390/ijms23084281 (PMC9030618; doi:10.3390/ijms23084281)
Supplement: Supplementary file 1 [file ijms-23-04281-s001.zip › ijms-1616825-supplementary.pdf]

***APOB* CRISPR-Cas9 engineering in hypobetalipoproteinemia: a promising tool for functional studies of novel variants**

SUPPLEMENTARY MATERIALS

Supplemental Table S1: Description of Primers used in this study

Step	Exon	Name	Sequence	PCR Size
**PCR and sequencing**	apoBE9	Bex9New-F	ATTTGGCCAAGGTTTGAAAGTT	396
**PCR and sequencing**	apoBE9	Bex9New-R	CTTTTCCCCTGTGCTGATACTC	
**CRISPR**	apoBE9	crRNA	mA*mA*mGrCrUrGrGrUrUrArCrUrGrArGrCrUrGrArG/crRNA-XT/	
**CRISPR**	apoBE9	ssODN	/5Alex488N/A*A* AAT ATC CAG AGAGCT AAT CTC TTC AAT AAG CGG GTTACC GAG CTG CGA GGC CTC AGT GATGAA GCA GTC ACA TCT C*T*C	
**ddPCR**	apoBE17	capoB_2647_F	CTGGAGCTGGATTACAGTTGCAAA	113
**ddPCR**	apoBE18	capoB_2760_R	AGGGTTTTGCCACCAGTTCAGC	
**ddPCR**	B2M	Hs_B2M_1_SG	Qiagen (Hilden, Germany) Geneglobe reference number : QT00088935	98

Supplemental Figure S1: Sanger sequencing of HuH7 engineered cells.

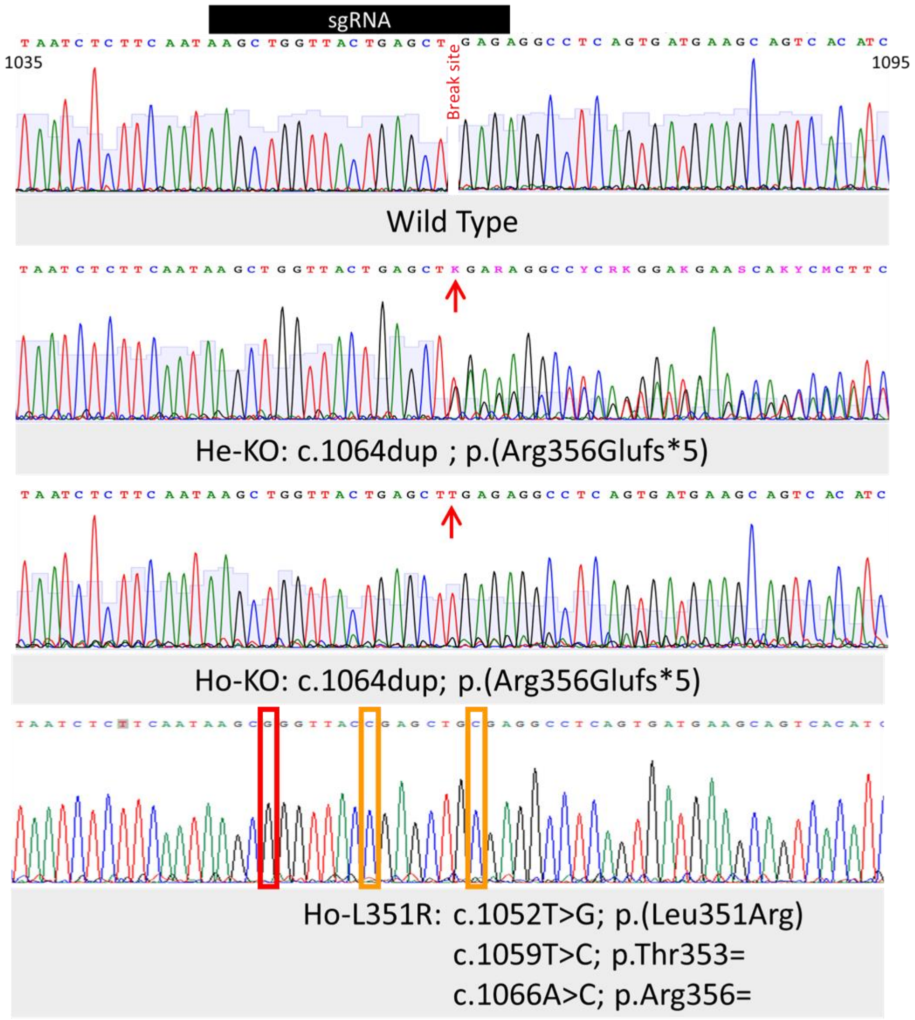

Supplemental Figure S2: Cell sorting process using flow cytometry.

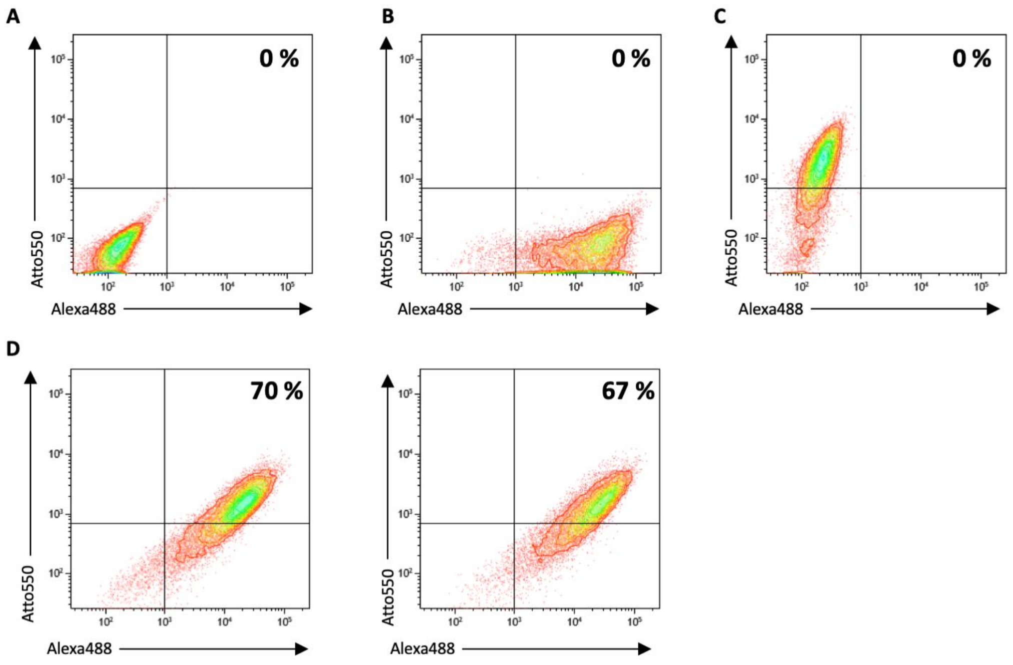

Cell sorting protocol and the position of double positive cells (upper right quadrant) were first defined by analyzing non-transfected cells (dotplot A), cells transfected only with ssODN tagged with AF488 (dotplot B), ssODN tagged with Atto550 (dotplot C) by flow cytometry. Representative examples of 2 transfection experiments are shown (Panel D). Only cells included in the upper right quadrant (i.e. double positive cells), representing around 70% of total cells, were cell sorted for further experiments.
